# Persistent Hematuria and Proteinuria in a Patient with SLE Nephritis

**DOI:** 10.34067/KID.0000001025

**Published:** 2026-04-30

**Authors:** Tripti Singh, Sarah E. Panzer, Shivani Garg

**Affiliations:** Department of Medicine, University of Wisconsin-Madison, Madison, Wisconsin

**Keywords:** Fabry disease, genetics and development, GN, lupus nephritis

## Abstract

**Figure:**
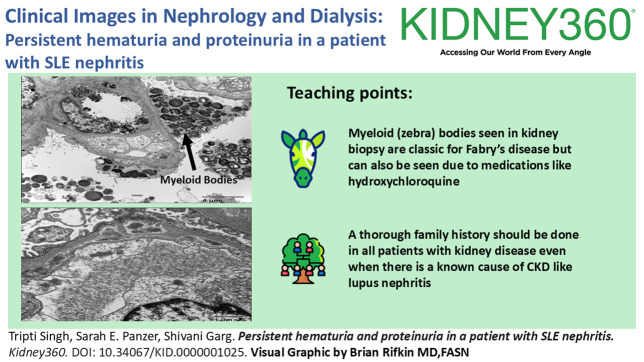

## Case Discussion

**Figure 1:**
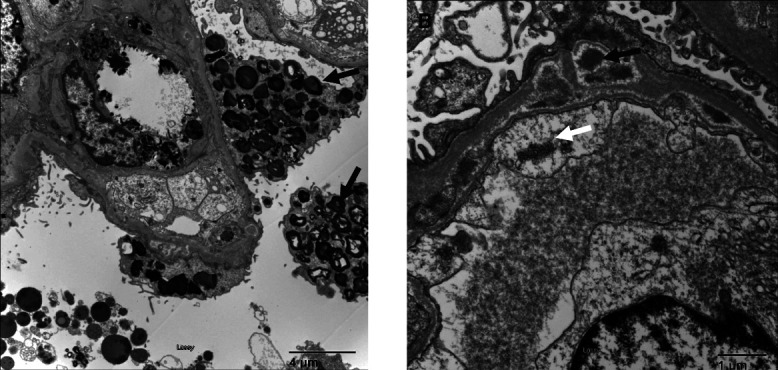
**Electron microscopy of kidney biopsy.** (A) Arrows point to zebra or myeloid bodies seen in the podocytes in Fabry disease. (B) Black arrow points to electron-dense subepithelial deposits and white arrow points to tubuloreticular lesions seen in lupus nephritis.

A 33-year-old woman with childhood-onset SLE and lupus nephritis presented with persistent hematuria and proteinuria despite being on various immunosuppressive agents including mycophenolate, azathioprine, rituximab, tacrolimus, belimumab, and chronic steroids over 10 years. Her current medications included azathioprine, tacrolimus, obinutuzumab, prednisone, lisinopril, and dapagliflozin without significant improvement in her proteinuria. Given the refractory disease despite multiple treatment for lupus nephritis, a repeat kidney biopsy was performed. Kidney biopsy showed large amount of zebra or myeloid bodies in podocytes (Figure 1A, arrow) and subepithelial electron dense deposits (Figure 1B, black arrow) and tubuloreticular inclusion (Figure 1B, white arrow) suggestive of lupus nephritis. Myeloid bodies are characteristic of Fabry disease, but can be seen as a medication side effect, such as hydroxychloroquine. However, 5 years ago, hydroxychloroquine was stopped because of concern for early maculopathy secondary to hydroxychloroquine. Hence, myeloid bodies were likely not related with hydroxychloroquine. A thorough family history revealed that patient's maternal grandfather and maternal uncle died of kidney disease in mid 40–50's. A genetic analysis was pursued and was positive for single X-linked variants in alpha-galactosidase gene leading to deficiency of lysosomal *α*-galactosidase A (*α*-Gal A), associated with Fabry disease. The patient was evaluated by genetics and urine ceramide trihexoside and serum globotriaosylsphinogosine levels, which checked and were elevated. The patient had a cardiac, ophthalmologic, and dermatologic evaluation and did not have any cardiac, eye, or skin involvement with Fabry disease. She did not have neurologic involvement, with no evidence of acroparesthesia. The patient was started on enzyme replacement therapy for treatment of Fabry disease.

## Discussion

This case highlights the importance of a thorough family history of kidney disease even in patients with a known cause of kidney disease such as lupus nephritis in our patient.^1^ Because the patient had a known cause of CKD and proteinuria diagnosed on initial kidney biopsy, family history of Fabry disease was missed until the zebra bodies were identified in the podocytes on second kidney biopsy. A thorough family history was taken when the patient was not responding to treatment of lupus nephritis. *α*-Gal A is required for breakdown of terminal galactose from globotriaosylceramide, thus leading to its accumulation in lysosomes causing myeloid bodies seen in podocyte of our patient's kidney biopsy leading to proteinuria and hematuria rather than hydroxychloroquine toxicity.^2^

This cause also highlights the importance of multidisciplinary care in the complex patients like this patient, which involved the discussion of kidney biopsy with the pathologist, discussion with rheumatologist and the geneticist both for diagnosis and management of her kidney disease. As Fabry disease is an X-linked disease, it typically does not present in women as clinical disease. It was thought to be causing kidney disease in our patient as she continues to have proteinuria and CKD despite her lupus in remission. The geneticist also thought that it was the inflammatory milieu of lupus nephritis which caused kidney involvement with Fabry disease as her heart and eyes did not have any involvement with Fabry disease.

## Teaching Points

- Myeloid bodies seen in kidney biopsy are classic for Fabry disease but can also be seen because of medications like hydroxychloroquine.
- A thorough family history should be done in all patients with kidney disease even when there is a known cause of CKD like lupus nephritis.
